# Meprin β expression modulates the interleukin‐6 mediated JAK2‐STAT3 signaling pathway in ischemia/reperfusion‐induced kidney injury

**DOI:** 10.14814/phy2.15468

**Published:** 2022-09-18

**Authors:** Shaymaa Abousaad, Faihaa Ahmed, Ayman Abouzeid, Elimelda Moige Ongeri

**Affiliations:** ^1^ Department of Kinesiology College of Health and Human Sciences, North Carolina A&T State University Greensboro North Carolina USA

**Keywords:** interleukin‐6, ischemia/reperfusion, JAK2/STAT3 signaling, meprin β, metalloproteinase

## Abstract

Meprin metalloproteinases have been implicated in the pathophysiology of ischemia/reperfusion (IR)‐induced kidney injury. Previous in vitro data showed that meprin β proteolytically processes interleukin‐6 (IL‐6) resulting in its inactivation. Recently, meprin‐β was also shown to cleave the IL‐6 receptor. The goal of this study was to determine how meprin β expression impacts IL‐6 and downstream modulators of the JAK2‐STAT3‐mediated signaling pathway in IR‐induced kidney injury. IR was induced in 12‐week‐old male wild‐type (WT) and meprin β knockout (βKO) mice and kidneys obtained at 24 h post‐IR. Real‐time PCR, western blot, and immunostaining/microscopy approaches were used to quantify mRNA and protein levels respectively, and immunofluorescence counterstaining with proximal tubule (PT) markers to determine protein localization. The mRNA levels for IL‐6, CASP3 and BCL‐2 increased significantly in both genotypes. Interestingly, western blot data showed increases in protein levels for IL‐6, CASP3, and BCL‐2 in the βKO but not in WT kidneys. However, immunohistochemical data showed increases in IL‐6, CASP3, and BCL‐2 proteins in select kidney tubules in both genotypes, shown to be PTs by immunofluorescence counterstaining. IR‐induced increases in p‐STAT‐3 and p‐JAK‐2 in βKO at a global level but immunoflourescence counterstaining demonstrated p‐JAK2 and p‐STAT3 increases in select PT for both genotypes. BCL‐2 increased only in the renal corpuscle of WT kidneys, suggesting a role for meprins expressed in leukocytes. Immunohistochemical analysis confirmed higher levels of leukocyte infiltration in WT kidneys when compared to βKO kidneys. The present data demonstrate that meprin β modulates IR‐induced kidney injury in part via IL‐6/JAK2/STAT3‐mediated signaling.

## Graphical abstract

Meprin metalloproteases are implicated in acute and chronic kidney disease. The current study shows that meprin beta expression associates with high levels of interleukin‐6 proteins in ischemia/reperfusion‐induced kidney injury. Downstream of IL‐6, meprin beta impacts the JAK‐STAT3 mediated signaling pathway and the apoptotic genes CASP3 and BCL‐2.

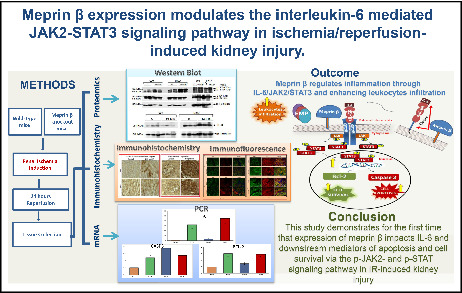

**New and Noteworthy Annotation**

This study demonstrates for the first time that expression of meprin β by proximal tubule cells and leukocytes impacts IL‐6 and downstream mediators of apoptosis and cell survival via the p‐JAK2‐ and p‐STAT signaling pathway in IR‐induced kidney injury. Previous in vitro data showed proteolytic processing of IL‐6 by meprin β, resulting in inactivation of the IL‐6. This study confirms that proteolytic processing of IL‐6 by meprin β impacts inflammation in vivo.

## 1 INTRODUCTION

Ischemia/reperfusion (IR) is a major cause of acute kidney injury (AKI), with adverse clinical effects that include tubulointerstitial inflammation (Thurman et al., 2006). Meprins metalloproteinases are abundantly expressed in the brush border membranes (BBMs) of kidney proximal tubules and small intestines (Sterchi et al., 2009). Meprins are also expressed in leukocytes (monocytes and macrophages; Sun et al., 2009), suggesting a role in the immune response. Meprin have been implicated in the pathophysiology of IR‐induced kidney injury. Meprin β‐deficient mice showed a significant protection against renal IR injury, indicating that meprin expression exacerbates IR‐induced kidney injury (Bylander et al., 2008). However, the mechanisms by which meprins modulate kidney injury are not fully understood. In vitro studies showed that meprin β proteolytically processes interleukin‐6 (IL‐6), leading to inactivation of IL‐6 (Keiffer & Bond, 2014). It was recently reported that meprins also cleave the IL‐6 receptor (IL‐6R; Arnold et al., 2017). Thus, existing data suggest that meprins could modulate IL‐6‐mediated inflammation. IL‐6 binds to its membrane bound receptor (mbIL‐6R) activating the classic IL‐6 signaling pathway or the soluble form of the receptor (sIL‐6R) activating the IL‐6 trans‐signaling pathway (Kaur et al., 2020). In both signaling cascades, the IL‐6/IL‐6R complex activates the membrane‐bound gp130 dimer, which in turn activates the Janus Kinase2‐Signal Transducer and Activator of Transcription3 (JAK2/STAT3; Heinrich et al., 1998; Kaur et al., 2020; Mascareno et al., 2001; Schindler & Strehlow, 1999). This activation of STAT3 by tyrosine phosphorylation leads to translocation of phosphorylated STAT3 (p‐STAT3) into the nuclei, and transcription of several genes that include the pro‐apoptosis genes, B‐cell lymphoma/leukemia 2 (BCL‐2; Horiguchi et al., 2002), anti‐apoptosis genes, and cysteine‐aspartic acid protease 3 (Caspase3, CASP3; Zhao et al., 2020). However, it is not known whether meprin β cleavage of IL‐6 in vivo and subsequent inactivation of IL‐6 modulate downstream mediators of the IL‐6 signaling pathway in kidney tissue. Meprin β expression on macrophages and its ability to cleave extra‐cellular matrix (ECM) proteins suggest that meprin B could enhance leukocyte infiltration (Bedau et al., 2017; Bylander et al., 2008; Crisman et al., 2004; Yura et al., 2009), thus indirectly contributing to inflammation in the kidneys subjected to IR injury. The goal of the current study was to determine how meprin β expression mediates inflammation via modulation of IL‐6 levels and downstream mediators of IL‐6 signaling pathway in mice kidneys subjected to IR injury.

## 2 MATERIALS AND METHODS

### 2.1 Experimental animals

Wild‐type (WT) and meprin β knockout (βKO) male mice on a C57BL/6 background were used. The WT mice express both meprin α and meprin α, and therefore have all three mrprin protein isoforms while the βKO mice are deficient in two meprin protein isoforms, meprin B (β–β) and the heterodimeric form of meprin A (α‐β). The βKO mice were generated by the laboratory of Judith Bond, Pennsylvania State University. The βKO mice were bred at the Laboratory Animal Resource Unit (LARU) of North Carolina A&T State University (NC A&T). Age‐matched WT mice were purchased from Charles River Laboratories (Wilmington, MA). The mice were housed in groups of up to five mice per standard cage and were fed a standard rat chow (Purina) and water ad libitum with exposure to a 12:12 h light–dark cycle. All the animal protocols for this study were approved by the NC A&T Institutional Animal Care and Use Committee (IACUC).

### 2.2 Induction of kidney injury

We induced kidney injury in 12‐week‐old male mice by clamping the renal pedicle of the kidney for 27 min as previously described (Ahmed et al., 2020) followed by 24 h reperfusion. The contralateral kidney was not clamped and served as the control for each mouse. The mice were then euthanized by CO_2_ asphyxiations and kidney tissues harvested for proteomics and immunohistochemical analysis. A minimum of four mice were per genotype were euthanized at 24 h post‐IR.

### 2.3 Processing of kidney tissues

The harvested kidneys were de‐capsulated and sections of each kidney processed appropriately for protein extraction, RNA extraction, or paraffin embedding and subsequent immunohistochemistry. For protein extraction, kidney sections were wrapped in aluminum foil and snap‐frozen in liquid nitrogen, then stored at −80°C. For immunohistochemistry, 2 mm mid‐section tissue samples were stored in Carnoy's fixative (60% ethanol/30% chloroform/10% acetic acid) overnight at 4°C, then transferred to 70% ethanol at 4°C until processed for paraffin embedding. Paraffin embedding and cutting tissue sections onto slides were performed at the Wake Forest University Pathology Laboratory. The kidney tissue samples for RNA extraction were stabilized and stored in RNALater® solution (Invitrogen Cat# AM7021) for 24 h at 4°C. After 24 h, the RNALater® was aspirated and tissues were stored at −80°C until used for RT‐PCR analysis.

### 2.4 Assessment of kidney injury

**FIGURE 1:**
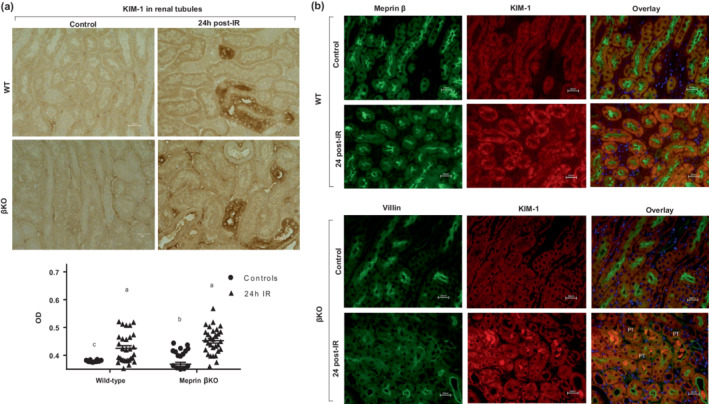
Immunohistochemical staining for kidney injury marker 1 (KIM‐1). (a) Ten non‐overlapping fields for tubular and 10 non‐overlapping fields of renal corpuscle sections from each kidney were imaged at 60× magnification and analyzed in a blinded manner. Relative optical density (ODs) values were quantified (*n* = 3 mice/group) and analyzed using Image J analysis Software. The OD data were analyzed by two‐way ANOVA. (b) Immunofluorescence counterstaining of KIM‐1 (red) and meprin β (green) in wild‐type (WT) and villin (green) in meprin β knockout (βKO) kidneys to determine KIM‐1 protein localization as an indicator of kidney injury. DAPI (blue) was used to stain the nuclei. There were significant increases in KIM‐1 in select proximal tubules at 24 h post‐IR in both genotypes (*p* < 0.0001), confirming kidney injury.

Because injury was not induced in the contralateral kidney, blood samples could not be used for biochemical assessment of kidney function. Instead, sections of each kidney were subjected to immunohistochemical staining for kidney injury molecule‐1 (KIM‐1), an established kidney injury biomarker. Immunohistochemical data from 3 mice per group showed that the expression of KIM‐1 increased significantly (*p* ≤ 0.0001) in select tubules for kidneys subjected to IR for both genotypes relative to their control counterparts, confirming injury in kidneys subjected to IR **(**Figure 1a).

### 2.5 RNA extraction and cDNA synthesis

Kidney tissues were disrupted using a tissue homogenizer (Bead Mill 4 Homogenizer, Thermo Scientific Cat# 15‐340‐164) and total RNA from control and ischemic kidneys were isolated using the Qiagen RNeasy Mini Kit (Qiagen Cat# 74106) according to manufacturer's guideline. Concentration and purity were determined at 260/280 and 260/230 using a spectrophotometer (Spectrophotometer NanoDrop 2000, Thermo Scientific Cat# 13400519). Denatured RNA was reverse transcribed into cDNA in a 20 μl reaction volume using High‐Capacity cDNA Reverse Transcription Kit with RNase Inhibitor (Thermo Fisher Cat# 4368814). Reverse transcription was performed at 37°C for 90 min, 85°C for 3 min, followed by quick chilling on 4°C and obtained cDNA stored at −20°C until subsequent amplification.

### 2.6 Real‐time PCR analysis

Two‐steps RT‐PCR reactions were performed with QuantiFast SYBR® Green PCR Reagents (Qiagen Cat# 204056) according to the manufacturer's instructions using Bio‐Rad's Multiplate™ 96‐Well PCR Plates. The qPCR cycling conditions were 50°C for 2 min, 95°C for 10 min followed by 40 cycles of a two‐step amplification program (95°C for 15 s and 58°C for 1 min). At the end of the amplification, melting curve analysis was applied using the dissociation protocol from the Sequence Detection System to exclude contamination with non‐specific PCR products. Oligonucleotides for all genes were designed as mouse‐specific primer pairs obtained from Integrated DNA Technologies (IDTDNA) (Corlvielle, IO). Oligonucleotides sequences of the primer sets are: (i) IL‐6, Forward: GTT CTC TGG GAA ATC GTG GA, Reverse: TGT ACT CCA GGT AGC TAT GG (Ma et al., 2021); (ii) BCL‐2, Forward: GCC TTT TTC TCC TTT GGC GG, Reverse: AAG AGT GAG CCC AGC AGA AC (Damodaran et al., 2020); (iii) CASP3, Forward: GAG CTT GGA ACG GTA CGC TA, Reverse: CCG TAC CAG AGC GAG ATG AC (Al‐Megrin et al., 2020). The mRNA expressions of target genes were presented as a fold change relative to control samples of WT kidneys. Data were normalization using the mRNA of housekeeping gene GAPDH: Forward: AGG TCG GTG TGA ACG GAT TTG, Reverse: GGG GTC GTT GAT GGC AAC A (Chen et al., 2017) and analyzed via the 2^–ΔΔCt^ method (Schmittgen & Livak, 2008).

### 2.7 Protein extraction from kidney tissues

Protein Extraction from kidney tissues utilized previously described protocols (Ahmed et al., 2020; Niyitegeka et al., 2015; Ongeri et al., 2011). Briefly, kidneys were homogenized in 9 volumes of ice‐cold buffer (0.02 mM HEPES pH 7.9, 0.015 mM NaCl, 0.1 mM Triton‐X 100, 0.01 mM SDS, 1 mM Na3VO4) with protease and phosphatase inhibitors. RIPA buffer was used to obtain protein lysates from sections of the kidney tissue. Protein concentrations in each sample were determined via the Bradford protein assay method using Bio‐Rad's protein assay reagent (Bio‐Rad). All the extracted proteins were stored in aliquots at −80°C until analyzed by western blot.

### 2.8 Western blot analysis

Western blot analysis was used to evaluate the protein levels of IL‐6, phospho‐STAT3 (Tyr^705^) (p‐STAT3), phospho‐JAK2 (Tyrosine ^1007+1008^, Y1007 + Y1008) (P‐JAK2), Caspase3 (CASP3), and BCL‐2 in the kidney tissues using previously described protocols (Ahmed et al., 2020; Niyitegeka et al., 2015; Ongeri et al., 2011). Kidney Proteins (45–90 μg) were subjected to electrophoresis on 8%–12% polyacrylamide gels and transferred to nitrocellulose membrane. Non‐specific bindings were blocked by incubating in 5% fat‐free milk in Tris‐buffered saline with 0.1% Tween (Chen et al., 2017) (TBS‐T) for 1 h at room temperature. Nitrocellulose membranes were incubated with primary antibodies at room temperature for 1 h or overnight at 4°C as follows: IL‐6 (Abcam Cat# ab9324, RRID:AB_307175) diluted 1:1000, p‐STAT3 (Cell Signaling Technology Cat# 9145, RRID:AB_2491009) diluted 1:500, STAT3 (Cell Signaling Technology Cat# 30835, RRID:AB_2798995) diluted 1:2000, p‐JAK2 (Abcam Cat# ab32101, RRID:AB_775808) diluted 1:300, CASP3 (Cell Signaling Technology Cat# 9662, RRID:AB_331439) diluted 1:1000, BCL‐2 (Cell Signaling Technology Cat# 15071, RRID:AB_2744528) diluted 1:1000, and Anti‐β tubulin (Origene Cat# TA301569) diluted 1:7000. The tubulin served as a loading control. Secondary antibody, either goat anti‐mouse(Bio‐Rad Cat# 172–1011, RRID:AB_11125936) or anti‐rabbit IgG (Bio‐Rad Cat# 170–6515, RRID:AB_11125142) dilution 1: 10,000 were added onto the membrane and incubated for 1 h at room temperature or overnight at 4°C. The membranes were exposed to chemiluminescence substrates (Thermo Scientific Cat# 34577) and developed on X‐ray film. The protein band intensities were determined by densitometry using Image Studio™ Lite Software (Version 2.5.2). To obtain the relative optic densities (relative ODs) for each protein, the ODs for each protein band were normalized to the ODs of β‐tubulin for the same sample. The ODs for phosphorylated proteins were normalized to their corresponding non‐phosphorylated total proteins ODs.

### 2.9 Immunohistological analysis

Immunohistochemical staining was used to evaluate the protein expression of KIM‐1, IL‐6, p‐STAT3, p‐JAK2, CASP3, and BCL‐2 using previously described protocols (Ahmed et al., 2020; Niyitegeka et al., 2015; Ongeri et al., 2011). In summary, slides were deparaffinized by immersing in Xylene 2 times for 5 min each, 100% Ethanol 2 times for 3 min each, 95% Ethanol 2 times for 3 min each, and distilled water 1 time for 5 min. Slides were then exposed to antigen unmasking via boiling in 10 mM sodium citrate buffer, pH 6.0, for 10 min. The slides were then immersed in methanol (MeOH) quench buffer (25% of 30% H2O2 in MeOH) for 20 minutes to quench endogenous peroxidase activity. Slides were washed for 5 min in PBS‐T (1% BSA and 0.3% Triton‐X‐100), then incubated in 1% normal goat serum in PBS buffer at room temperature for 1 h in a humidified chamber in order to block the non‐specific binding sites. Slides were then incubated in primary antibodies diluted in PBS buffer with 2.5% normal goat serum overnight at 4°C or at room temperature for 1 h. Antibodies used were; rabbit polyclonal anti‐KIM‐1 antibodies (Abcam Cat# ab47635, RRID:AB_882998) diluted 1:100, mouse monoclonal anti‐IL‐6 (Abcam Cat# ab9324, RRID:AB_307175) diluted 1:1000, rabbit polyclonal anti‐p‐STAT3 (Cell Signaling Technology Cat# 9145, RRID:AB_2491009) diluted 1:500, rabbit monoclonal anti‐p‐JAK2 (Abcam Cat# ab32101, RRID:AB_775808) diluted 1:1000, rabbit monoclonal anti‐CASP3 (Cell Signaling Technology Cat# 9662, RRID:AB_331439) diluted 1:500 and mouse monoclonal anti‐BCL‐2 (Cell Signaling Technology Cat# 15071, RRID:AB_2744528) diluted 1:400, rabbit polyclonal anti‐CD45 diluted 1:200 (Abcam Cat# ab10558, RRID:AB_442810), rabbit monoclonal anti‐F4/80 (Abcam Cat# ab111101, RRID:AB_10859466) diluted 1:200. After that, slides were washed in PBS 3 times for 5 min each. Slides were incubated for 30 min in a secondary antibody solution (BPS buffer with 2% universal biotinylated secondary antibody and 2% normal goat serum), and then washed in PBS 2 times for 5 minutes each. For standard immunostaining, we used the Vectastain® Elite® ABC Universal Kit Protocol (Vector Laboratories Cat# PK‐6200, RRID: AB_2336826) following the manufacturer's instruction. The tissue sections were evaluated for IL‐6, p‐STAT3, p‐JAK2, CASP3, BCL‐2, CD45 and F4/80 using light microscope (KEYENCE Corporation of America) and imaged using BZ‐X700 analysis Software. Ten non‐overlapping fields for tubular and 10 non‐overlapping fields of renal corpuscle were imaged at 60× magnification from each kidney section and analyzed in a blinded manner. To determine staining intensity levels for IL‐6, p‐STAT3, p‐JAK2, CASP3 and BCL‐2, calibrated 8–bit images based on the quantified OD standard were evaluated for optical density values (ODs) via Image J analysis Software (ImageJ/Fiji 1.46). For evaluation of leukocyte infiltration, kidney sections were probed with anti‐CD45 and anti‐F4/80 antibodies and the number of positive staining cells were counted in 10 non‐overlapping tubulointerstitial sections and 10 renal corpuscles per kidney in a double‐blinded manner.

### 2.10 Immunofluorescence staining

Immunofluorescence counterstaining was used to determine the localization of the proteins of interest according to the previously described protocol (Ahmed et al., 2020), with meprin and villin as proximal tubule biomarkers. Briefly, slides were deparaffinized by immersing in Xylene 3 times for 5 min each, 100% Ethanol 2 times for 10 min each, 95% Ethanol 2 times for 10 min each, and distilled water 2 times for 5 min each. Slides then were exposed to antigen unmasking via boiling in 10 mM sodium citrate buffer, pH 6.0, for 10 min. In order to block the non‐specific binding sites, slides were incubated in blocking buffer of PBS with 5% normal goat serum and 0.3% Triton X‐100 for 1 h at room temperature. Slides then were incubated in primary antibodies diluted in a dilution buffer of PBS with 0.3% Triton X‐100 and 1% BSA at same dilution levels as described in the previous immunohistological analysis section. Sections were counterstained overnight at 4°C or at room temperature for 1 h with polyclonal goat anti‐mouse meprin β antibodies for WT and with villin for βKO sections: mouse monoclonal anti‐villin antibodies (Santa Cruz Biotechnology Cat# sc‐58,897, RRID:AB_2304475) diluted 1:200 and goat anti‐mouse polyclonal meprin β antibodies (R and D Systems Cat# AF3300, RRID:AB_2143451) diluted 1:200. The slides were then rinsed three times in PBS for 10 min each and incubated for 1 h at room temperature in fluorophore‐conjugated secondary antibodies diluted in same dilution buffer at 1:1000: chicken polyclonal anti‐rabbit, Alexa Fluor® 488 (Invitrogen, Cat# A‐21441, RRID:AB_2535859) for KIM‐1, p‐STAT3, p‐JAK2 and CASP3; chicken monoclonal anti‐mouse, Alexa Fluor® 488 (Invitrogen Cat# A‐21200, RRID:AB_2535786) for IL‐6 and BCL‐2; donkey polyclonal anti‐mouse, Alexa Fluor® 647 (Abcam Cat# ab150107, RRID:AB_2890037) for villin and chicken polyclonal anti‐goat, Alexa Fluor® 488 (Invitrogen, Cat# A‐21467, RRID:AB_141893) for meprin β. Diluted 4,6‐diamidino‐2‐phenylindole (DAPI) (Vector Laboratories Cat# SK‐4100, RRID:AB_2336382) was used for nuclear staining (1:1000). To prevent fluorescence signal from fading, all slides were covered by coverslips with prolong anti‐fade reagent (Life Technologies) and allowed to dry at room temperature overnight. Tissue sections were evaluated for expression and localization using a BZ‐X700 Series all‐in‐one fluorescence microscope (KEYENCE Corporation of America) and imaged using BZ‐X700 analysis software at 60× magnification.

### 2.11 Statistical analysis

Data analysis of mRNA expression of the target genes were performed for each group versus the WT control group. All data were analyzed by two‐way ANOVA with Tukey's pair‐wise comparisons using GraphPad 7.0 Prism Software (GraphPad). Data are presented as mean ± SEM. *p* ≤ 0.05 were considered statistically significant.

## 3 RESULTS

The levels of the kidney injury biomarker, KIM‐1, increased in both genotypes for kidneys subjected to IR but not in control counterparts (Figure 1a), confirming that our surgical procedure induced kidney injury. Immunofluorescence counterstaining with proximal tubules biomarkers (meprin β in WT and villin in βKO kidneys) showed that high KIM‐1 expression level was predominantly in the proximal tubules (PTs) and not in distal tubules (DTs) (Figure 1b). Interestingly, we also observed KIM‐1 shed in the lumen of PTs in βKO kidney sections, suggesting KIM‐1 excretion and clearance into the urine after kidney insult in AKI as previously shown by others (Sohotnik et al., 2013; Peng et al., 2012).

### 3.1 Meprin β deficiency associated with increased IL‐6 protein levels in kidney tissue at 24 h post‐IR

**FIGURE 2:**
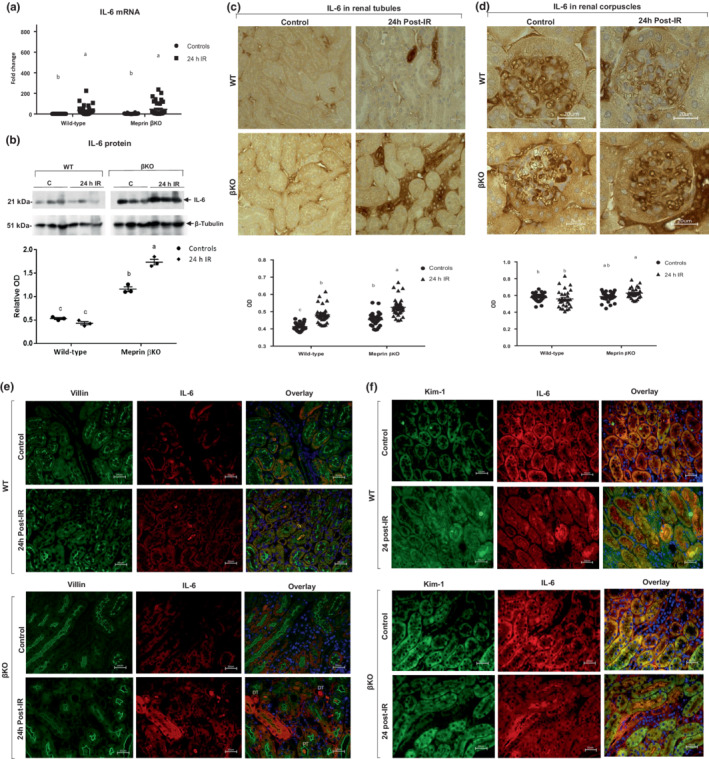
IL‐6 mRNA and protein expression in kidney tissue. (a) Relative mRNA levels for IL‐6. Values for IL‐6 mRNA levels were presented as fold change relative to control WT kidneys. Each value represents the mean ± SEM of triplicate combinations from 4 mice per group. Data were and analyzed by two‐way ANOVA. a–c means values with different letters are significantly different. (b) Representative immunoblots of kidney IL‐6 proteins. The protein bands in each lane represent samples from individual kidneys. The relative ODs were calculated by normalizing the ODs of IL‐6 to the OD for β‐tubulin in the same sample. Data are means ± SEM from 3 mice per treatment group. (c) Immunohistochemical staining for IL‐6 in kidney tubules. (d) Immunostaining for IL‐6 in renal corpuscles. Ten non‐overlapping fields for tubular and 10 non‐overlapping fields of renal corpuscle sections from each kidney were imaged at 60× magnification. Relative ODs were quantified (*n* = 3 mice/group) and analyzed by two‐way ANOVA. A–c means values with different letters are significantly different. There was a significant increase in IL‐6 mRNA and protein in select tubules in both genotypes (*p* < 0.01). (E) Localization of IL‐6 and KIM‐1 in kidney tubules. Immunofluorescence counterstaining of IL‐6 (red) and the proximal tubule marker, villin (green); (F) Co‐localization of KIM‐1 (green) and IL‐6 (red). DAPI (blue) was used to stain the nuclei.

To determine the impact of meprin β expression/activity on IL‐6 levels in vivo, mRNA and protein expression of IL‐6 were evaluated in kidney tissue at 24 h‐post‐IR. Real‐time PCR data showed a significant increase in IL‐6 mRNA levels in both WT (*p* ≤ 0.01) and βKO (*p* ≤ 0.0001) mice subjected to IR when compared to the counterpart control kidneys (Figure 2a). Western blot data showed that IL‐6 protein levels significantly increased in βKO kidneys (*p* ≤ 0.0001) but not in WT counterparts at 24 h post‐IR (Figure 2b), suggesting meprin β‐mediated decreases in IL‐6. Interestingly, immunohistochemical staining of kidney sections for IL‐6 showed significant increases in IL‐6 expression in select kidney tubules for both genotypes but no significant change in the renal corpuscles (Figure 2c,d). To identify the localization of increased KIM‐1 and IL‐6 expression in kidney tissues, we used immunofluorescence counterstaining with proximal tubule biomarker, villin in both WT and βKO kidneys. In WT, IL‐6 expression was observed in both PTs, which express meprin β and DTs, which lack meprin β. We also observed increases in IL‐6 levels in the lumen of PTs only in WT kidney sections at 24 h post‐IR. On the other hand, we observed increases in IL‐6 levels in the lumen of both PTs and DTs in βKO kidneys, suggesting IL‐6 excretion and clearance into the urine at 24 h post‐IR **(**Figure 2e). Our data also showed that IL‐6 expression was positively associated with KIM‐1 and thus kidney injury in several tubules in both genotypes subjected to IR **(**Figure 2f). The data further suggest that western blot analysis is not sensitive in evaluating changes in IL‐6 protein expression patterns as the increase is not global but in select tubules.

### 3.2 Meprin β deficiency associated with increased renal p‐JAK2 protein expression at 24 h post‐IR

**FIGURE 3:**
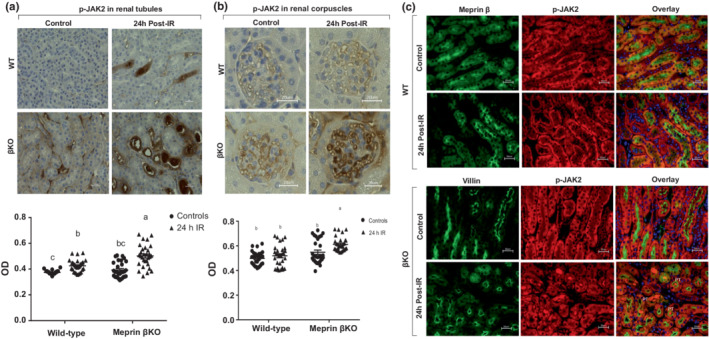
p‐JAK2 protein expression in kidney tissue. (a) Immunohistochemical staining for phosphorylated JAK2^Y1007+Y1008^ (P‐JAK2) protein in tubules. (b) Immunostaining for p‐JAK2 in renal corpuscles. Relative ODs were quantified for 10 non‐overlapping fields of tubular and 10 non‐overlapping fields of renal corpuscle sections for each kidney. OD data were quantified (*n* = 3 mice/group) and analyzed by two‐way ANOVA. a–c Mean values with different letters are significantly different (*p* < 0.01). (c) Immunolocalization for p‐JAK2 (red) in kidney tubules. Meprin β (green) and villin (green) were used as PT biomarkers in WT and βKO respectively. There were significant increases in p‐JAK2 levels in PTs in both genotypes and in renal corpuscle of βKO kidneys only.

To determine whether meprin β expression affects downstream modulators of the IL‐6 signaling pathway, levels of p‐JAK2^Y1007+Y1008^ were evaluated using western blot analysis and immunohistochemical staining approaches. Phosphorylated protein levels of Janus kinase on Tyrosine 1007 and 1008 (p‐JAK2^Y1007+Y1008^) could not be detected using western blot analysis. However, light microscopy and analysis of the immunostaining showed that p‐JAK2 levels significantly increased in select tubules of both WT (*p* = 0.005) and βKO (*p* ≤ 0.0001) kidneys at 24 h post‐IR when compared to counterpart control kidneys **(**Figure 3a). However, in renal corpuscles, p‐JAK2 levels significantly increased only in the βKO (*p* ≤ 0.0001) and not in WT kidney sections subjected to IR when compared to their corresponding controls **(**Figure 3b). Immunofluorescence counterstaining with proximal tubule biomarkers, showed that IR‐induced increases in p‐JAK2 levels occurred in the PTs and not in DTs for both genotypes with comparable baseline expression levels in the PTs of control kidneys of both genotypes **(**Figure 3c). Additionally, immunofluorescence staining showed increase p‐JAK2 levels in the lumen of PTs only in βKO group, suggesting increased release of p‐JAK2 into filtrate and subsequently into urine.

### 3.3 Meprin β deficiency associated with increase in p‐STAT3 α and β levels at 24 h post‐IR

**FIGURE 4:**
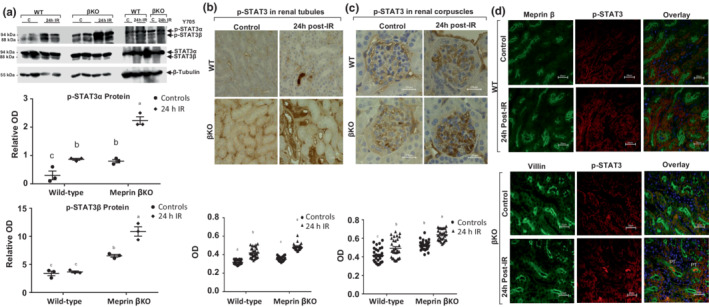
p‐STAT3 proteins in kidney tissue. (a) Representative immunoblots of the phosphorylated STAT3 (Tyr^705^) spliceforms, 94‐kDa p‐STAT3α^Y705^ and 88‐kDa p‐STAT3β^Y705^ proteins. The relative optic densities (ODs) were calculated by normalizing the ODs of p‐STAT3α^705^ and p‐STAT3β^705^ to the OD of their corresponding non‐phosphorylated proteins levels STAT3α^Y705^ and STAT3β^Y705^ which were in turn normalized to β‐tubulin in the same blot. Data are mean ± SEM from 3 mice per group. (b) Immunohistochemical staining with p‐STAT3 protein in tubules. (c) Immunostaining for p‐STAT3 in renal corpuscles. Ten non‐overlapping fields for tubular and 10 non‐overlapping fields of renal corpuscle sections were imaged at 60X magnification from each kidney. OD data were quantified (*n* = 3 mice/group) and analyzed by two‐way ANOVA. a–d mean values with different letters are significantly different (*p* < 0.01). (d) Immunolocalization of p‐STAT3 (red) in kidney tubules. Meprin B (green) in WT and villin (green) in βKO were used as proximal tubule markers and DAPI (blue) was used to stain the nuclei. There were significant increases in protein levels of p‐STAT3α^705^ in both genotypes and p‐STAT3β^Y705^ in βKO with increase total p‐STAT3 in tubules and renal corpuscle of both genotypes.

To determine whether meprin β expression affects the levels of transcription factor signal transducer and activator of transcription 3 (STAT3), a downstream modulator of the IL‐6 signaling pathway, total STAT3 and the phosphorylated STAT3 on Tyrosine 705 (p‐STAT3^Y705^) proteins were evaluated using western blot analysis. Two isoforms for both forms were detected, STAT3‐α and p‐STAT3‐α^Y705^ (at 94 kDa) and STAT3‐β andpP‐STAT3‐β^Y705^ (at 88 kDa). Immunoblot analysis showed that baseline protein expression of both isoforms of p‐STAT3 were significantly lower (*p* ≤ 0.0001) in WT when compared to βKO kidneys **(**Figure 4a). Additionally, the 94‐kDa p‐STAT3‐α^Y705^ increased significantly in both βKO (*p* ≤ 0.0001) and WT (*p* ≤ 0.0232) kidney tissues subjected to IR when compared to control kidneys. However, p‐STAT3‐β^Y705^ increased only in βKO mice subjected to IR (*p* ≤ 0.0001) when compared to control mice but not in WT kidneys. On the other hand, levels of non‐phosphorylated 94‐kDa STAT3‐α and the 88‐kDa STAT3‐β proteins were comparable in all groups. Increased p‐STAT3^Y705^ protein levels was confirmed using light microscopy, with immunostaining intensity for p‐STAT3^Y705^ being significantly increased (*p* ≤ 0.0007) in select tubules and in renal corpuscles of both genotypes for kidneys subjected to IR when compared to control kidneys (Figure 4b,c). When compared to βKO kidneys, the p‐STAT3 baseline levels were lower in WT kidneys. Immunofluorescence counterstaining showed that the levels of p‐STAT3 proteins were high in PTs of both genotypes at 24 h post‐IR **(**Figure 4d). However, accumulation of p‐STAT3 in the lumen of PTs were observed only in βKO kidneys at 24 h post‐IR. Individual interstitial cells (presumed to be resident macrophages) stained positively for p‐STAT3^Y705^ in both genotype at 24 h post‐IR sections. The levels of p‐STAT3^Y705^ and expression patterns were comparable in PTs and DTs in both control and IR kidneys.

### 3.4 Meprin β deficiency increased the levels of pro‐apoptotic protein, CASP3, in proximal tubules at 24 h post‐IR

**FIGURE 5:**
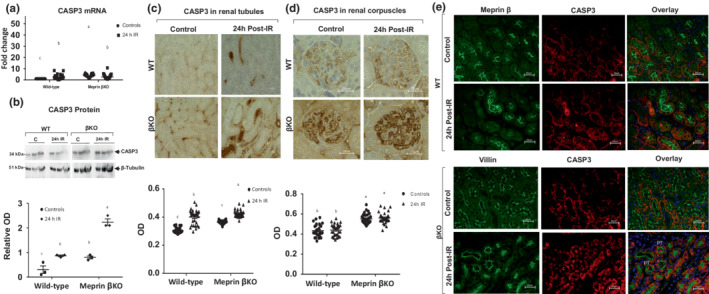
CASP3 mRNA and proteins in kidney tissue. (a) CASP3 mRNA levels. Values for mRNA levels are expressed as fold change relative to the wild‐type (WT) control group. Each value represents the mean ± SEM of triplicate combinations from 4 mice per group. Data were analyzed by two‐way ANOVA. a–c means values with different letters are significantly different. (b) Representative immunoblots of the CASP3 proteins. The protein bands represent samples from individual kidneys. The relative optic densities (ODs) were calculated by normalizing the ODs of CASP3 to the OD for β‐tubulin in the same sample. Data are means±SEM from 3 mice per group. (c) Immunohistochemical staining for CASP3 in kidney tubules. (d) Immunostaining staining for CASP3 in renal corpuscles. OD data were quantified (*n* = 3 mice/group)and analyzed by two‐way ANOVA for 10 non‐overlapping fields for tubular and 10 non‐overlapping fields of renal corpuscle sections from each kidney (imaged at 60× magnification). a–d means values with different letters are significantly different (*p* < 0.01). (e) Immunolocalization of CASP3 proteins (red) in kidney tubules. Meprin β (green) in wild‐type (WT) and villin (green) in βKO were used as PT markers and DAPI (green) was used to stain the nuclei. There was a significant increase in CASP3 mRNA and proteins in both genotypes.

To determine whether meprin β expression impacts downstream apoptotic targets of the IL‐6 signaling pathway, mRNA and protein levels of CASP3 were evaluated in kidney tissue. Data from RT‐PCR analysis showed a significant increase in CASP3 mRNA levels in both WT (*p* ≤ 0.001) and βKO (*p* ≤ 0.05) kidneys at 24 h post‐IR relative to their control counterpart kidneys. However, the baseline mRNA levels for CASP3 were significantly lower (*p* ≤ 0.0001) in WT when compared to βKO kidneys **(**Figure 5a). Western blot analysis showed that protein levels for CASP3 (detected at 35 kDa) significantly increased (*p* ≤ 0.0001) only in βKO kidneys subjected to IR compared to control kidneys **(**Figure 5b). Interestingly, the baseline protein expression of CASP3 was significantly lower (*p* ≤ 0.0001) in WT when compared to βKO kidneys. Immunohistochemical staining coupled with evaluation by light microscopy showed a significant increase (*p* ≤ 0.0001) in CASP3‐staining intensity in select tubules of both genotypes subjected to IR compared to their controls with a higher base level in βKO **(**Figure 5c). However, levels of CASP3 were comparable in the glomerular sections of both genotypes at 24 h post‐IR when compared to their control kidneys (Figure 5d). Immunofluorescence staining showed high CASP3 expression in PTs and DTs of WT kidneys at 24 h post‐IR **(**Figure 5e). However, in βKO kidneys subjected to IR, CASP3 expression increased only in PTs (Figure 5e). Additionally, CASP3 levels increased in the lumen of PTs in both genotypes at 24 post‐IR. These data suggested that meprin deficiency was associated with increased levels of pro‐apoptotic activity of CASP3 in response to IR.

### 3.5 Meprin β deficiency is associated with increase anti‐apoptotic protein, BCL‐2, at 24 h post‐IR

**FIGURE 6:**
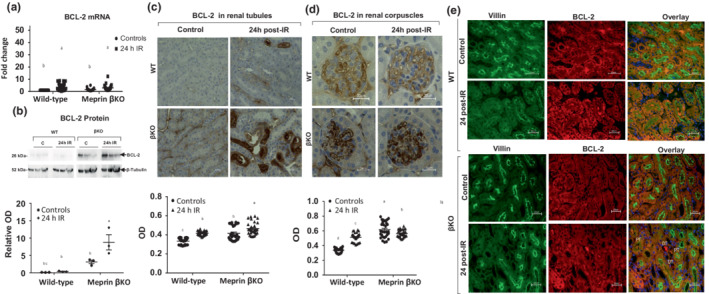
BCL‐2 mRNA and proteins in kidney tissue. (a) BCL‐2 mRNA. The values for mRNA levels are expressed as fold change relative to the wild‐type (WT) control group. Each value represents the mean ± SEM of triplicate combinations from 4 mice per group. Data were analyzed by two‐way ANOVA. a–c mean values with different letters are significantly different. (b) Representative immunoblots of the BCL‐2 proteins. The protein bands represent samples from individual kidneys. The relative optic densities (ODs) were calculated by normalizing the ODs of BCL‐2 to the OD for β‐tubulin in the same sample. Data are presented as mean ± SEM from 3 mice/group. (c) Immunohistochemical staining for BCL‐2 in tubules. (d) Immunostaining staining for BCL‐2 in renal corpuscle of kidney tissue. Ten non‐overlapping fields for tubular and 10 non‐overlapping fields of renal corpuscle sections were imaged at 60× magnification from each kidney section (*n* = 3 mice/group) and analyzed in a blinded manner. a–c mean values with different letters are significantly different (*p* < 0.01). (e) Immunolocalization of BCL‐2 proteins. Villin (green) was used as a PT marker and the DAPI (blue) was used to stain the nuclei. There were significant increases in BCL‐2 mRNA levels for both genotypes at 24 h post‐IR. However, BCL‐2 proteins increased only in βKO kidneys subjected to IR.

To determine whether meprin β expression impacts downstream anti‐ apoptotic targets of the IL‐6 signaling pathway, levels of BCL‐2 mRNA and proteins in kidney tissue were evaluated. RT‐PCR analysis showed IR‐induced increases in BCL‐2 mRNA levels in both WT (*p* ≤ 0.0001) and βKO (*p* ≤ 0.005) kidneys when compared to the counterpart control kidneys (Figure 6a). However, western blot data showed that total BCL‐2 protein levels (detected at 26 kDa) significantly increased (*p* = 0.0330) in the βKO only and not in WT kidneys subjected to IR **(**Figure 6b). Immunohistochemical analysis showed that BCL‐2 expression levels were significantly higher in select kidney tubules of both βKO (*p* ≤ 0.0001) and WT (*p* = 0.0025) kidneys subjected to IR when compared to their control counterparts (Figure 6c). Furthermore, BCL‐2 expression in the renal corpuscle was significantly higher in WT kidneys subjected to IR (*p* ≤ 0.0001) but not in βKO kidneys (Figure 6d). Counterstaining of BCL‐2 with proximal tubule markers (meprin β in WT and villin in βKO) showed that IR‐induced increases in BCL‐2 occur in both PTs and DTs (Figure 6e). Additionally, BCL‐2 secretion into the lumen was observed in both PTs and DTs tubules in βKO, but only on PTs of WT kidneys.

### 3.6 Meprin β increased the levels of leukocytes infiltration, in tubulointerstitium and renal corpuscles at 24 h post‐IR

**FIGURE 7:**
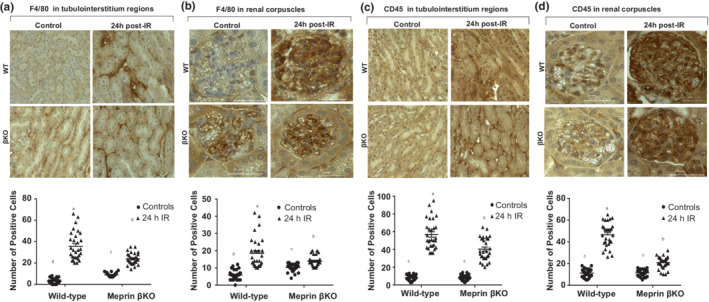
Leukocyte infiltration in kidney tissue. (a) Immunohistochemical staining for F4/80 in tubulointerstitium. (b) Immunostaining staining for F4/80 in renal corpuscle of kidney tissue. (c) Immunohistochemical staining for CD45 in tubulointerstitium. (d) Immunostaining staining for CD45 in renal corpuscle of kidney tissue. Ten non‐overlapping fields for tubulointerstitium and 10 non‐overlapping fields of renal corpuscle sections were imaged at 60× magnification from each kidney section (*n* = 3 mice/group). The positive stained cells were counted manually in a double blinded manner and the data subjected to 2‐way ANOVA. a–c means values with different letters are significantly different (*p* < 0.0001). The data show that meprin β enhanced leukocyte infiltration in both tubulointerstitium and renal corpuscles at 24 h post‐IR.

To determine whether meprin β expression impacts leukocytes infiltration which exacerbate inflammation, the leukocytes staining for F4/80 (macrophages marker) and CD45 (myeloid marker) were evaluated in 10 non‐overlapping tubulointerstitial sections and 10 renal corpuscles per kidney at 24 h post‐IR using standard immunohistochemical staining approaches. Immunohistochemical staining with F4/80 showed significant increases (*p* ≤ 0.0001) in the number of F4/80 positive stained cells in tubulointerstitium regions (Figure 7a) for both genotypes subjected to IR when compared to their control counterparts. Similarly, levels of F4/80 positive cells increased in renal corpuscles of both WT (*p* ≤ 0.0001) and βKO (*p* ≤ 0.05) kidneys subjected to IR when compared to control kidneys **(**Figure 7b). The same pattern was observed for when tubulointerstitium and renal corpuscles were evaluated for CD45 positive stained cells. Data showed that CD45 positive cells increased (*p* ≤ 0.0001) in tubulointerstitium regions (Figure 7c) and in renal corpuscles (Figure 7d) of both genotypes compared to their control counterparts at 24 h post‐IR. However, the number of positively staining cells for both leukocytes markers, F4/80 and CD45, were significantly higher (*p* ≤ 0.0001) in meprin β‐expressing mice (WT) when compared to meprin β‐deficient mice (βKO) subjected to IR, suggesting a role for meprin β in enhancing leukocyte infiltration in IR‐induced kidney injury.

## 4 DISCUSSION

IR is the leading cause of AKI and is associated with high morbidity and mortality rates (Patidar et al., 2022; Yali et al., 2022). Inflammation plays a central role in the progression of AKI (Han et al., 2020; Vázquez‐Carballo et al., 2021) and kidney injury induced by IR (Meng et al., 2020). In AKI, IL‐6 signaling is a key link for local and systemic inflammation (Joseph et al., 2020; Rahn & Becker‐Pauly, 2021; Shang et al., 2020). In the IL‐6 “classic signaling pathway” a pro‐inflammatory pathway, the epithelial cells membrane‐bound IL‐6 receptor (mbIL‐6R) binds to gp130 receptor to activate a downstream signaling cascade (Ebihara et al., 2011; Grigoryev et al., 2008; Malchow et al., 2011; Rose‐John & Heinrich, 1994; Scheller et al., 2011). On the other hand, when IL‐6 binds to its soluble receptor (sIL‐6R), the IL‐6/sIL6R complex binds to the membrane‐bound gp130 dimer to form a complex that activates the IL‐6 “trans‐signaling pathway”. The IL‐6 trans‐signaling is an anti‐inflammatory pathway (Rose‐John, 2017; Scheller et al., 2011) that plays a protective role by promoting repair processes in IR‐induced AKI (Lemay et al., 2000; Yoshino et al., 2003). The IL‐6 trans‐signaling pathway is dominant in cells that lack mbIL‐6R expression (Kaur et al., 2020; Rose‐John, 2017; Su et al., 2017). Meprin metalloproteinases have been implicated in the pathophysiology of kidney injury. Meprins are abundantly expressed in the BBM of kidney proximal tubules, and redistributed from BBMs of kidney proximal tubules to the cytoplasm and basolateral compartments of proximal tubule cells as an active shed form in IR‐induced AKI (Bylander et al., 2008). The redistribution allows meprin β to closely interact with proteins present in the cytosolic and basolateral cell compartments. Meprins are also expressed by leukocytes (monocytes and macrophages) suggesting a role in the immune response. More importantly, meprins have been shown to proteolytically process several proteins that modulate inflammation in vitro and in vivo. The membrane‐bound meprin β, proteolytically processes IL‐6, leading to inactivation of IL‐6 in vitro (Keiffer & Bond, 2014). It was recently reported that the membrane‐bound form of meprin β also cleaves the membrane‐bound IL‐6 receptor (mbIL‐6R) (Armbrust et al., 2021), leading to activation of the classic IL‐6 signaling on the mbIL‐6R expressing cell. In contrast, proteolytic release of the soluble form, sIL‐6R activates the trans‐signaling pathway on adjacent cells that do not express mbIL‐6R (Arnold et al., 2017; Su et al., 2017). As proximal tubule epithelial cells and macrophages do not express mbIL‐6R (Su et al., 2017), the IL‐6 pathway in these cells must be activated via IL‐6 trans‐signaling.

Data from the present study showed that in IR‐induced kidney injury, meprin β regulates expression of IL‐6. While data from RT‐PCR showed that IL‐6 mRNA expression levels increased in both genotypes subjected to IR, elevation in IL‐6 protein levels was correlated with meprin β deficiency in βKO mice subjected to IR. It is likely that the low levels of IL‐6 proteins observed in meprin‐expressing mice, despite the increases in IL‐6 mRNA levels, is due to the proteolytic processing of newly synthesized IL‐6 proteins by meprin β. These findings are consistent with data from previous studies; reinforcing the hypothesis, that meprin β modulates inflammation by processing and inactivating IL‐6 (Atreya & Neurath, 2005; Banerjee & Bond, 2008; Keiffer & Bond, 2014). The inflammatory mediators, such as IL‐6, contribute to the pathogenesis of tubular injury by mediating exfoliation of epithelial cells (Glynne et al., 2001; Wangsiripaisan et al., 1999). Data from the current study show that IL‐6 expression correlates with kidney injury in select tubules in both genotypes subjected to IR. Proximal tubules, which express meprins, are more susceptible to IR‐induced kidney injury when compared with distal tubular cells which are deficient in meprins (Weinberg et al., 1991). Additionally, proximal tubule cells interact with other resident cells of the renal cortex in producing or responding to co‐stimulatory cytokines (Yard et al., 1992). Immunofluorescence counterstaining with proximal tubule markers, show that IL‐6 expression increased primarily in meprin β‐expressing kidneys at 24 h post‐IR. This suggests that meprin‐mediated release of IL‐6 into filtrate and subsequently into urine, is partly responsible for the increased urinary levels of IL‐6 after IR‐induced injury (Bylander et al., 2008; Kwon et al., 2003).

We further investigated the effect of meprin β expression on the levels of the two main downstream modulators of the IL‐6 signaling pathway, JAK2 and STAT3. Previous studies reported that IL‐6 activates the JAK/STAT signaling pathway (Heinrich et al., 1998; Kaur et al., 2020; Mascareno et al., 2001; Schindler & Strehlow, 1999) which serves as a potential target for early intervention in IR‐induced acute renal failure (Yang et al., 2008). The IL‐6/JAK2/STAT3 axis plays roles in various biological functions, including immune regulation, lymphocyte growth and differentiation, oxidative stress (Garbers et al., 2018; Kang et al., 2019), cell proliferation, differentiation, cell migration and apoptosis (Ihle, 1996; Schindler & Strehlow, 1999). Activation of the JAK2/STAT3 cascade starts with the Janus kinase (JAK2) phosphorylation (p‐JAK2). Immunohistochemical analysis of kidney tissue from the current study showed that phosphorylated JAK2^Y1007^ increased in select kidney tubules of mice subjected to IR for both genotypes. Subsequently, JAK2 phosphorylates and activates the signal transducers and activators of transcription 3 (STAT3). STAT3 plays an important role in cytokine‐mediated induction of acute‐phase response (Abualsunun & Piquette‐Miller, 2018). Activation of STAT3 (dependent upon tyrosine phosphorylation), showed a rapid increase in injured renal tubule cells (Talbot et al., 2011), after IR injury (Arany et al., 2012; Ogata et al., 2012). Phosphorylation of STAT3 is the main regulator of IL‐6 target gene expression (Ihw et al., 2012; Jain et al., 1999). In previous studies, two STAT3 spliceforms were identified as short forms of STAT3 (STAT3β), which is missing the 55 C‐terminal amino acid residues of the long form (STAT3α) and has seven additional amino acid residues at its C terminus, with distinct functions for each isoform (Chakraborty et al., 1996; Schaefer et al., 1995). STAT3α normally has higher expression levels compared to STAT3β and acts as a pro‐ and anti‐inflammatory factor based on the activating signal (Hodge et al., 2005; Hutchins et al., 2013). On the other hand, STAT3β acts as a suppressor of systemic inflammation (Zhang et al., 2019) as well as a significant transcriptional regulator that has direct actions in modulating STAT3α activation after IL‐6 stimulation (Ihw et al., 2012). Additionally, induction of a splicing switch toward the STAT3β isoform leads to apoptosis and cell‐cycle arrest (Musteanu et al., 2010). However, at the phosphorylation level, absence of cytokine stimulation enhanced phosphorylation of STAT3α isoform (Hevehan et al., 2002). Our western blot data showed that phosphorylated STAT3α increased in both genotypes. However, phosphorylated STAT3β increased only in βKO at 24 h post‐IR. Taken together, our results provide compelling evidence that meprin β mediated IL‐6 response to IR‐induced renal injury is a STAT3β‐isoform‐specific effect. Furthermore, our immunohistochemical results demonstrated that IR induced a significant increase in p‐STAT3 in tubules and glomerular of both genotypes.

In addition to mediating tubule‐interstitial injury, meprins expressed in macrophages can mediate glomerular inflammation. A variety of cytokines are expressed by resident mesangial cells or by infiltrating leukocytes in renal corpuscles during the process of glomerular injury (Kanai et al., 2001), and mediate their inflammatory response via STAT3 activation (Zhang et al., 2005). For example, IL‐6 produced by macrophages induced mesangial proliferative glomerulonephritis (Horii et al., 1993). Activation of IL‐6/STAT3 signaling pathway in macrophages plays a key role in chemokine production from macrophages (Zhang et al., 2013) and involved in M1/M2 macrophage polarization (Yin et al., 2018). In addition, phosphorylation of STAT3 is a critical event associated with the status of macrophages activation (Matsukawa et al., 2003; Takeda & Akira, 2000, 2001; Welte et al., 2003). As JAK/STAT is the main intracellular signaling pathway of IL‐6 cytokine, it is likely that both JAK2 and STAT3 are activated also in the renal corpuscles during IR‐induced AKI. Data from the current study show that JAK2/STAT3 signaling was upregulated in the glomerular of mice with IR‐induced injury. On the other hand, the expression levels of CASP3 and BCL2 were not significantly elevated in the glomerular, which suggests that JAK2/STAT3 signaling could be activated prior to the increase of these exacerbating factors. Several studies indicated to the role of JAK/STAT signaling pathway in the renal corpuscles in other renal disease models (via STAT3), such as in experimental nephrotic syndrome, unilateral ureteral obstruction (Li et al., 2007; Pang et al., 2010), and Alport syndrome (Yokota et al., 2018). Furthermore, phosphorylated STAT3 (tyr^705^) proteins dimerize and translocate into the nucleus to regulate transcription of anti‐apoptosis gene, BCL‐2 (Horiguchi et al., 2002) and pro‐apoptosis gene (CASP3) (Zhao et al., 2020). Several previous studies showed that expression of BCL‐2 and CASP3 increased in IR injury (Domitrović et al., 2014; Kim et al., 2010; Lan et al., 2016; Sari et al., 2020; Vinuesa et al., 2008). In apoptosis, CASP3 (35 kDa) is activated by cleavage into cleaved a 17 kDa CASP3 fragment. However, the 17 kDa CASP3 was not detected by western blot analysis of kidney tissue from either genotype, suggesting that there is no significant apoptosis at 24 h post‐IR. Future studies will be done to determine the CASP3 cleavage at later time points. Data from immunolblots and immunohistological analysis in the current study showed meprin β mediates downstream IL‐6‐ apoptotic effects via increased CASP3 and BCL‐2 levels in IR‐induced kidney injury, which could be supported by the previously reported dual effect of CASP3 and inducing cellular responses other than apoptosis (Lamkanfi et al., 2007). For example, CASP3 was shown to play important role in T and B lymphocyte proliferation by acting as checkpoints to control their cell cycle and selective cleavage of the supressors or inducers of their cell cycle machinery (Launay et al., 2005). Additionally, CASP3 was shown to have a strict proteolytic activity on selected substrates (Lamkanfi et al., 2007; Launay et al., 2005) and controling cell survival (Franchi et al., 2003). Therefore, under some conditions, CASP3 seems to be cytoprotective rather than cytotoxic, but this dual effect is not fully understood. The high expression of CASP3 and BCL‐2 proteins found in our study, indicate that cell apoptosis in IR tissues might be controlled by the balance of these two pro‐apoptotic and anti‐apoptotic factors. Our data also showed that BCL‐2 increased in the renal corpuscles of WT kidneys at 24 h post‐IR, suggesting a role for meprin β expressed in leukocytes.

In addition to direct modulation of inflammatory pathways, meprin β could enhance leukocyte infiltration (Herzog et al., 2019) via cleavage of extracellular matrix proteins (ECM) proteins such as collagen IV, laminin, nidogen‐1, and fibronectin (Kaushal et al., 1994), cytoskeletal proteins (actin and villin) (Ongeri et al., 2011), and tight junction proteins (E‐cadherin and occludin) (Bao et al., 2013; Huguenin et al., 2008). This hypothesis is supported by data from the current study with the number of cells positive for F4/80 (EGF‐like module‐containing mucin‐like hormone receptor‐like 1, macrophages marker) and CD45 (lymphocyte common antigen, myeloid marker) being significantly higher in WT kidneys in both tubulointerstitium regions and renal corpuscles at 24 h post‐IR. These results are aligned with data from previous studies (Bedau et al., 2017; Bylander et al., 2008; Crisman et al., 2004; Yura et al., 2009). Furthermore, inflammatory macrophage accumulation adjacent to tubular cells (tubulointerstitium regions) showed to be associated with tubular apoptosis (Lange‐Sperandio et al., 2003; Tesch et al., 1999). IL‐6 trans‐signaling also involves in leukocyte trafficking and infiltration (Jones & Rose‐John, 2002; Kaplanski et al., 2003), controls leukocyte apoptosis and the expression of inflammatory chemokines and adhesion molecules (Atreya et al., 2000; Chen et al., 2004; Hurst et al., 2001; Marin et al., 2001; McLoughlin et al., 2003; Modur et al., 1997; Romano et al., 1997).

In summary, our data suggest that proteolytic processing of IL‐6 by meprin β plays an important role in modulating IL‐6 expression and influences the downstream signal transduction/pathway mediated via JAK2/STAT3 in IR‐induced kidney injury. The data provide evidence for meprin β modulation of IL‐6/JAK2/STAT3 signaling and their convergence to activate the downstream target genes of IL‐6 signaling in IR‐induced kidney injury. Taken together, data from the current study provide new insights on how meprin β regulates the pathophysiology of kidney injury through the IL‐6/JAK2/STAT3 signaling pathway.

## FUNDING INFORMATION

These studies were supported by funding from the National Institutes of Health (NIH) Award numbers SC1GM3102049 and R35GM141537 to Elimelda Moige Ongeri. Faihaa Ahmed was supported by NIH grant number T32 AI007273.
